# Religious Values in Clinical Practice are Here to Stay

**DOI:** 10.1007/s10943-018-0715-y

**Published:** 2018-10-17

**Authors:** Alex Kappel Kørup, Jens Søndergaard, René dePont Christensen, Connie Thurøe Nielsen, Giancarlo Lucchetti, Parameshwaran Ramakrishnan, Klaus Baumann, Eunmi Lee, Eckhard Frick, Arndt Büssing, Nada A. Alyousefi, Azimatul Karimah, Esther Schouten, Andreas Schulze, Inga Wermuth, Niels Christian Hvidt

**Affiliations:** 1 Research Unit of General Practice, Institute of Public Health, University of Southern Denmark, Odense, 5000 Denmark; 2 Department of Mental Health Service, University of Southern Denmark, Vejle, Denmark; 3 Department of Medicine, Federal University of Juiz de Fora, Avenida Eugênio de Nascimento s/n-Aeroporto, Juiz de Fora, 36038330 MG Brazil; 4 Graduate Theological, Union-University of California, 2400 Ridge Rd, Berkeley, CA 94709 USA; 5 AdiBhat Foundation, New Delhi, 110048 India; 6 Caritas Science and Christian Social Work, Faculty of Theology, Albert-Ludwig-University, 79085 Freiburg, Germany; 7 Research Centre Spiritual Care, Department of Psychosomatic Medicine and Psychotherapy, The University Hospital Klinikum rechts der Isar, Langerstr. 3, Munich, 81675 Germany; 8 Munich School of Philosophy, Kaulbachstr. 31, Munich, 80539 Germany; 9 Institute of Integrative Medicine, Faculty of Medicine, Witten/Herdecke University, Gerhard-Kienle-Weg 4, Herdecke, 58313 Germany; 10 College of Medicine, King Saud University (KSU), Riyadh, 11461 Saudi Arabia; 11 Department of Psychiatry, Faculty of Medicine, Universitas Airlangga, Dr. Soetomo General Hospital, Surabaya, East Java, Indonesia; 12 Department of Neonatology, University Hospital Munich, Marchioninistrasse 15, 80366 Munich, Germany; 13 Department of Child and Adolescent Psychiatry, Psychosomatics and Psychotherapy, University Hospital Munich, Munich, Germany; 14 Academy of Geriatric Cancer Research (AgeCare), Odense University Hospital, 5000 Odense, Denmark

**Keywords:** Religion, Value-neutrality, Clinical practice, Physicians, Medical ethics

## Abstract

Research to date has shown that health professionals often practice according to personal values, including values based on faith, and that these values impact medicine in multiple ways. While some influence of personal values are inevitable, awareness of values is important so as to sustain beneficial practice without conflicting with the values of the patient. Detecting when own personal values, whether based on a theistic or atheistic worldview, are at work, is a daily challenge in clinical practice. Simultaneously ethical guidelines of tone-setting medical associations like American Medical Association, the British General Medical Council and Australian Medical Association have been updated to encompass physicians’ right to practice medicine in accord with deeply held beliefs. Framed by this context, we discuss the concept of value-neutrality and value-based medical practice of physicians from both a cultural and ethical perspective, and reach the conclusion that the concept of a completely value-neutral physician, free from influence of personal values and filtering out value-laden information when talking to patients, is simply an unrealistic ideal in light of existing evidence. Still we have no reason to suspect that personal values, whether religious, spiritual, atheistic or agnostic, should hinder physicians from delivering professional and patient-centered care.

“God is dead. God remains dead. And we have killed him.” So wrote Nietzsche in 1882 referring to the God of Christian tradition. Yet religiosity is thriving, and the number of people affiliated with a religion is estimated to increase by 2.3 billion by the year 2060 to a total of 8.4 billion religious individuals on the planet, an increase of 3.5 percent points relative to the total population (Pew Research Center (2017) The Changing Global Religious Landscape).

Physicians and other medical professionals host a wide array of personal values in constant rearrangement throughout their lives. Major sources and influences include ideological, ethical, political, humanistic, religious and/or spiritual reflections, as well as one’s personal history and experiences. While Beauchamp and Childress in their renowned work “Principles of Biomedical Ethics” present four basic value-based principles to guide health professionals: (1) Respect for Autonomy, (2) Abstain from causing harm, (3) Principle of beneficence and (4) Principle of justice, they also state that information provided to patients not rarely must be free from the “entrenched values and goals of medical professionals” (Beauchamp and Childress 2001), hereby indicating the inherent challenges associated with the utilization of personal values in clinical practice. While some personal values of physicians can be acceptable, others can conflict with those of the patient. Most patients would agree that a good physician adheres to the above four principles, but patients are entitled to oppose the influence of other personal values of physicians where these are felt incompatible with those of the patient. In highly secular cultures, patients often expect physicians to leave personal religious or atheistic values out of the consultation completely. But whether this distance between physician and patient is advantageous is not clear. It has even been argued that a match of concordant values, including religious or spiritual orientation in the physician–patient relationship, could improve the quality of treatment (Peteet 2014).

A note must be made on why our focus in this viewpoint is mainly on religious values and not atheistic ones, as these secular values of physicians are equally important potential influencers on clinical practice, for good and for worse. Over the last 500 hundred years, modern evidence-based medicine has largely parted ways from its philosophical and religious roots, and many have argued that this secular shift has resulted in a lack of focus on spiritual and existential care (Sulmasy 2002). A physician having a secular stance may fail to fully understand a dying patient who openly declares that he is taking advice from a priest or imam on how his end-of-life treatment best favors his transition to after-life. Our focus on the religious values in this article, and not primarily on agnostic or atheistic values, is an attempt to address this reduced emphasis on value-based medical care. We also stress that our aim is not to favor any philosophy, theistic, atheistic or otherwise.

Over the recent years, the emphasis on physicians’ right to practice medicine according to their personal beliefs has emerged in influential ethical guidelines like the latest version of the American Medical Association (AMA) Code of Medical Ethics that states “physicians should have considerable latitude to practice in accord with well-considered, deeply held beliefs that are central to their self-identities” (Code of Medical Ethics of the American Medical Association 2016), and thus acknowledges respect for p*hysician* autonomy as well as that of their patients. Over the past decade, research has shown that physicians’ personal values, including religious, influence their (1) empathic relations with patients, (2) ethical standpoints and (3) understanding of own practice. Also religious physicians are more likely to discuss religious or spiritual issues with their patients (Farr A. Curlin et al. 2006), less likely to refer their patients to a mental health facility (Curlin et al., 2007), and more likely to accept clergy and pastoral professionals in the care of their patients (Daaleman and Frey 1998). They also have a higher prevalence of religious objections to physician-assisted suicide, terminal sedation and withdrawal of life support (Curlin et al. 2008); and are also less likely to report that they must disclose information about, or refer patients to, medical procedures to which they have personal religious objections (Curlin et al. 2007). Conversely, studies have shown that personal spirituality deepened empathy of the physicians and the sense of the existential needs of patients (Clark et al. 2003; Puchalski et al. 2009; Shepherd et al. 2017). Acknowledging and accepting the existence of this influence of personal beliefs into clinical practice calls for a reassessment of the ideal of value-neutrality.

Public health beliefs vary across cultures, and it is not uncommon that the explanation models for disease used by laymen (and often also their physicians) have deep religious or spiritual roots (McLaughlin and Braun 1998). From 3500BC to 500BC, shamans and priests-physicians mainly saw diseases of the body or mind as supernatural and sought to treat these through magical powers of incantations, laying-on-of-hands and in some cases herbal medicine (Koenig et al. 2012). Although Hippocrates and Aristotle founded modern evidence-based medicine and helped shape the basis of secular scientific thought in the years 460BC to 322BC (Sallam 2010), gods and evil spirits still explained the sickness of men through centuries to come. Still after the life of Jesus Christ, ideas of healing through prayer and miracle cures were generally accepted up through the Middle Ages and are still practiced in many churches today. In Europe, the Christian church became the leading authority in the caring for the sick (Ferngren 1992), and monasteries and hospitals were founded by the church on moral frameworks based on religious teachings, altruism and the tradition of the diaconate. Inspired by the Gospels, including the story of the Good Samaritan, the care and medicine practiced within these institutions were delivered as an extension of religious teachings.

Muslims might view a newly diagnosed cancer as the will of Allah and even as a method to connect with Allah (Rassool 2000), whereas in a Hindu context following the concept of Karma, the disease could be seen as a consequence of past failures to live according to the Dharma (Gupta 2010). In these rather clear cases, it would only be natural that the culturally accepted health beliefs were addressed in the clinical situation. But also in other less clear situations personal beliefs and values impact the lives of patients and ought to be acknowledged and addressed.

As physicians in most developed countries today are allowed to practice medicine congruent with their personal belief systems, they are still obliged to uphold the ethical norms of their profession “including fidelity to patients and respect for patient self-determination” (Code of Medical Ethics of the American Medical Association 2016). The British Medical Association (BMA) and the British General Medical Council (GMC) like AMA allow physicians to conscientiously object to procedures or treatments that are inconsistent with their personal belief system; insofar physicians respectfully refer patients to another physician. Both BMA and GMC argue for the importance of doctors to exercise restraint on the expression of own values or beliefs, where these might be detrimental to the interests of the patient. A common encouragement is to make any restrictions in practice due to religious beliefs clear to patients prior to engaging in a patient–physician relationship (Australian Medical Association Code of Ethics, revised, 2008; Code of Medical Ethics of the American Medical Association 2016; Good medical practice, GMC, 2013).

We cannot expect physicians to leave personal values not based solely on a common morality on the coat hanger when checking in at the office. Not only would this be practically impossible, but it would also weaken the integrity and autonomy of the physician. In a nation-wide study of American physicians’ religious values, 55% of physicians reported that their religious beliefs influenced their practice of medicine (Curlin et al. 2005), underlining the commonness of the influence of personal values in clinical practice, especially since other personal values like atheistic/agnostic, ideological and political values are not accounted for in this statistic.

Still every day physicians make decisions in companionship with, or even on behalf of, patients. Often these decisions are not straight forward textbook examples, but involve ethical dilemmas of varying complexity. Approaching these dilemmas physicians have to use their personal ethical skills, and not solely knowledge based on the medical curriculum. Often several norms and/or virtues must be weighed against each other, introducing a risk of shifting authority from a common morality to a particular morality biased by the personal values of the physician. It is therefore of great importance that physicians do not ignore how personal values might influence this shift in practiced morality. No matter the degree of self-awareness of the physicians, we question whether it is realistic to expect physicians themselves to be able to tell the difference between norms derived from one’s personal belief system over another? The morality taught by many organized belief systems is at its base not very distinct from the common morality described by Beauchamp & Childress, and thoughts derived thereof may not be experienced as religious or spiritual in essence. Often, religious and/or spiritual values are built upon this common morality making this a pluralistic and relativistic conglomerate of values. The values are taught, deliberately or through examples of role models, throughout childhood into adulthood, and thus become deeply integrated into the individual’s self. In this case, it would make little sense to try to delineate the intersecting borders between belief and self. From a psychotherapeutic perspective, beliefs and associated values have become a natural and fully accepted part of the individual’s self-image (i.e., egosyntonic), and thoughts and behavior derived thereof would not automatically give rise to any special attention compared to other thoughts and feelings.

We hereby call to mind that it is simply impossible to filter the impact of personal values (whether formed by atheism or by religiosity) out of the patient encounter. Rather, it is important to focus on the presence, function and impact of values in health care in order to secure the freedom and autonomy of both physician and patient values. In our opinion, the concept of a completely value-neutral physician, free from influence of personal values and filtering out value-laden information when talking to patients, is simply an unrealistic ideal in light of existing evidence. Still we have no reason to suspect that personal values should hinder physicians from delivering professional and patient-centered care.

We therefore argue, in contrary to Nietzsche’s statement, that at least in clinical practice God is not dead. Rather it seems the value-neutral physician is, and remains, dead.

Governments, health-care suppliers and tone-setting medical associations worldwide should accept this reality and work toward a future where the utilization of personal values is encompassed into the entire career chain of medical professionals, from the curriculum in medical school to clinical training of professionals.

Also further non-normative research in the influence of religious values in clinical practice is needed to continually build on our understanding of how personal values influence physician decision making, and not least patient experience and health outcome.
